# Canaliculitis Awareness

**DOI:** 10.4274/tjo.68916

**Published:** 2016-01-05

**Authors:** Melike Balıkoğlu Yılmaz, Emine Şen, Ebru Evren, Ufuk Elgin, Pelin Yılmazbaş

**Affiliations:** 1 Dr. Behçet Uz Children’s Disease and Surgery Education and Research Hospital, Ophthalmology Clinic, İzmir, Turkey; 2 Ulucanlar Eye Education and Research Hospital, Ankara, Turkey; 3 Başkent University Faculty of Medicine, Department of Microbiology, Ankara, Turkey

**Keywords:** Canaliculitis, canaliculotomy, conjunctivitis, curettage

## Abstract

**Objectives:**

To evaluate the demographic characteristics, treatment, and results of patients with canaliculitis.

**Materials and Methods:**

Medical records including the demographic characteristics, clinical findings, and treatment outcomes of patients diagnosed and treated for canaliculitis between September 2009 and March 2014 were analyzed retrospectively.

**Results:**

The median age of the 7 canaliculitis patients consisting of 4 women and 3 men was 49 (range 8-58) years. All patients had unilateral canaliculitis (on the right side in 2 and left side in 5 patients) and the inferior canaliculus was involved more frequently (71.4%). Epiphora, chronic conjunctivitis, a palpable and thickened canaliculus, and yellow discharge from the punctum were present in all cases. Actinomyces spp. was the most frequently cultured microorganism (75%). Dacryolith was observed in 6 patients. Canaliculotomy and dacryolith removal with canalicular curettage were performed, followed by medical treatment (topical penicillin 100,000 U/ml and oral ampicillin/sulbactam) for 10 days. Patients were followed up for a mean duration of 17.0±15.2 (range 3-46) months. Signs and symptoms resolved completely within a month. Epiphora recurred in the 36th month in a single patient and was treated with daily canalicular irrigation with antibiotics and there were no further symptoms during 10 months of follow-up after the recurrence.

**Conclusion:**

Canaliculitis is often overlooked and can be misdiagnosed. Every patient with chronic conjunctivitis and lacrimal infection should be examined carefully for canaliculitis.

## INTRODUCTION

Primary canaliculitis is a rare, chronic condition that develops with no underlying cause, usually due to actinomyces or staphylococci infection. It accounts for only 1.2-2% of all lacrimal disease.^1,2,3^ The clinical signs are punctal or canalicular edema, redness, and purulent discharge from the punctum when pressure is applied. Despite its clinical signs being very well defined, it can be easily overlooked and misdiagnosed.^4^ There are reports in the literature of diagnosis being delayed up to three years.^5^ Conservative treatment with topical antibiotic eye drops alone results in a high recurrence rate.^6,7^ Canaliculotomy and curettage of the dacryoliths are the gold standard in treatment.^4,8^ The aim of this study was to evaluate the demographic characteristics, treatments and outcomes of patients with canaliculitis.

## MATERIALS AND METHODS

Medical records including demographic characteristics, clinical findings, and treatment outcomes of patients diagnosed and treated for canaliculitis in the Oculoplasty Unit of the Ulucanlar Eye Education and Research Hospital between September 2009 and March 2014 were analyzed retrospectively. The study was approved by the institutional review board.

Seven patients referred by various doctors had been previously misdiagnosed and received inappropriate treatment. All cases were evaluated for potential coexisting eyelid diseases. All patients underwent surgical treatment consisting of canaliculotomy and dacryolith removal. Canaliculotomy was performed by making an incision in the affected canaliculus with a number 11 blade attached to a Bowman lacrimal probe. All dacryoliths and sulfur granules were carefully removed by curettage using a chalazion curette.

The samples were transported to the laboratory for microbiological analysis as soon as possible using anaerobic transport medium. For example, direct Gram staining revealed gram-positive, branching filamentous structures; cultures were made to ascertain the presence of aerobic and anaerobic bacteria and fungi. Columbia blood agar plates were incubated at 37 °C in anaerobic conditions for at least 5 days. Blood agar and MacConkey agar plates were incubated at 37 °C for 24-48 hours. Sabauroud dextrose agar plates were incubated at both 25 °C and 37 °C. Gram staining of the bacteria grown in the anaerobic environment revealed gram-positive branching bacilli, which were identified by biochemical tests. A diagnosis of Actinomyces canaliculitis was confirmed.^9^

The canaliculi were irrigated with an antibiotic. All patients were treated with hot compresses, 100,000 U/ml topical penicillin 8 times daily for 10 days, and systemic ampicillin/sulbactam, 750 mg orally twice a day for adults and 1 dose in the morning and 1/2 a dose in the evening of 400/57 mg/5 ml (1 dose) suspension for children. The canaliculi were allowed to heal without silicone intubation or reconstruction.

## RESULTS

**Figure 1:**
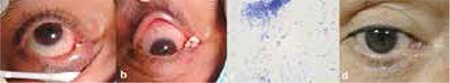
(a) Findings of purulent discharge from the right inferior punctum and hyperemia of the nasal conjunctiva in a patient with right inferior canaliculitis; (b) macroscopic appearance of the sulfur granules after inferior canaliculotomy and curettage; (c) 100x magnification of Gram staining showing infiltration of Actinomyces colonies; (d) patient’s appearance 6 months after surgery

**Figure 2:**
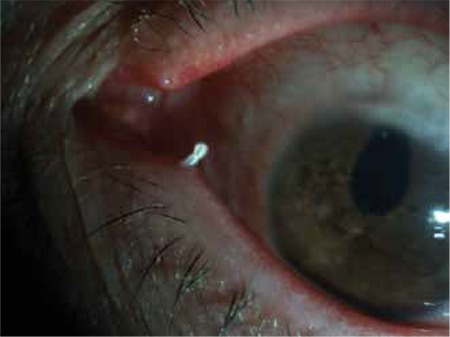
A patient with canaliculitis due to an eyelash entering the left inferior canaliculus

Seven patients were diagnosed with canaliculitis during the study period. Four of the patients were female, 3 were male, and the median age was 49 (range, 8-58) years. The mean follow-up time was 17.0±15.2 (range, 3-46) months. Four of the patients had been initially misdiagnosed with conjunctivitis, one with nasolacrimal duct obstruction (NLDO), and one with chalazion. One patient was found to have secondary canaliculitis due to an eyelash that entered the canaliculus during routine follow-up for glaucoma. This patient had also been previously treated for conjunctivitis. All cases were unilateral (two right, five left); the inferior canaliculus was affected more often (71.4%) (Table 1). Epiphora, chronic conjunctivitis, a palpable and thickened canaliculus, and yellow discharge from the punctum were present in all cases (Figure 1a). The median duration of symptoms was 9 (range, 1-36) months. Six patients had dacryoliths. Lacrimal lavage through the unaffected canaliculus was patent in all patients. Canaliculotomy was performed as surgical treatment. An incision was made in the inner palpebral area and mushroom-like whitish-yellow dacryoliths were removed (Figure 1b). After removing the dacryoliths from the canaliculi by curettage, all patients underwent medical treatment for 10 days (100,000 U/ml topical penicillin eight times a day and systemic ampicillin/sulbactam, 750 mg orally twice a day for adults or 1 dose in the morning and 1/2 dose in the evening of 400/57 mg oral suspension. Evaluation of the whitish-yellow dacryoliths revealed the presence of actinomycosis (Figure 1c). Actinomyces spp. were the most frequently cultured microorganism (75%). One patient was found to have secondary canaliculitis due to an eyelash entering the canaliculus (Figure 2). The patient had presented with eye redness and discharge to a primary care physician, who attempted to treat the condition believing it was conjunctivitis. The patient’s symptoms completely resolved with removal of the eyelash and topical antibiotic treatment. The patients were followed for a mean duration of 17.0±15.2 (range, 3-46) months. All patients’ signs and symptoms completely resolved within the first month (Figure 1d). For one patient, epiphora recurred in the 36th month; the patient was treated with canalicular irrigation with antibiotics daily for three days and then every other day for one week. The symptoms did not return during the 10 month post-recurrence follow-up period (46 months after surgery).

## DISCUSSION

The clinical signs of primary canaliculitis include a ‘pouting’ punctum, eyelid edema and erythema, mucopurulent discharge from the punctum when pressure is applied, and in some cases yellow dacryoliths called ‘sulfur granules’ in the punctum. Although these clinical signs are well defined, because the condition is rarely encountered it can easily be missed and misdiagnosed as conjunctivitis, mucocele, dacryocystitis, blepharitis or meibomian gland cyst, resulting in delayed diagnosis.^3,4,7,10^

Primary canaliculitis usually occurs with no underlying cause, although canalicular occlusion or diverticulum may precipitate infection in the canaliculus.^11^ It is usually unilateral and affects the inferior canaliculus, though there are reports in the literature of cases in the superior canaliculus.^3,10^ In our study the inferior canaliculus was involved in 71.4% of the cases.

Canaliculitis is most commonly seen in middle-aged and elderly patients, although 5- and 6-year old patients have also been reported in the literature.^10,12,13^ According to age, the annual incidence of canaliculitis per 100,000 population has been reported as 0.04 in the first decade, 0.27 in the second decade, 0.59 for the 40-59 age group, and 1.37 for the 60-79 age group.^14^ Similarly, the median age of the patients in our study was 49, and we had one 8-year-old patient. Park et al.^12^ reported a 5-year-old patient who underwent surgery after repeated probing and balloon dacryoplasty to treat congenital NLDO did not improve symptoms; canaliculitis was diagnosed during the surgery and curettage was performed. Park et al.^12^ believed that the patient’s previous surgeries may have created a predisposition to canaliculitis. Yaman et al.^10^ reported no history of any diseases or surgical procedures underlying their cases. Many investigators have reported that canaliculitis occurs more frequently in women.^3,4,6,11,13^ This is thought to be due to hormonal changes in menopause and reduced tear production disrupting the barriers that prevent infection.^4,6^ In our study, 4 of the 7 patients were women, but only one was in menopause. There have also been reports of secondary canaliculitis associated with the use of oral 5-fluorourasil in breast cancer treatment and more recently, due to trauma related to the insertion of punctal plugs.^15,16,17^ We found that one of our patients had secondary canaliculitis due to an eyelash entering the canaliculus. The patient’s symptoms resolved completely with removal of the eyelash and topical antibiotic treatment. Previously this patient had been unsuccessfully treated for what the physician believed was conjunctivitis.

Canaliculitis is a condition that can easily be diagnosed with a careful clinical examination, without the need for detailed examinations like dacryocystography.^3^ However, in cases that are uncertain, feeling the presence of dacryoliths in the canaliculus during nasolacrimal duct lavage can aid diagnosis.^11^ Without correct diagnosis and appropriate treatment, the condition recurs frequently. If patients with complaints of recurrent unilateral epiphora and discharge were started on topical antibiotics for conjunctivitis but their signs and symptoms return after a brief period of improvement, as occurred with our patients, a more careful examination of the canaliculi should be done with canaliculitis in mind for the differential diagnosis. Kaliki et al.^13^ reported a median diagnostic delay of 6 (range, 1-60) months in their series of 74 primary canaliculitis patients. The median symptom duration in our cases was 9 (range, 1-36) months.

Anand et al.^3^ emphasized that repeated forceful nasolacrimal lavage can push canalicular granules into the lacrimal sac and lead to NLDO, which increases the importance of early and accurate diagnosis. At the same time, canaliculitis can be mistaken for dacryocystitis or NLDO, as occurred with one of our patients. Observing patency to nasolacrimal duct lavage through the unaffected canaliculus is important in the differential diagnosis.

Of the agents involved in canaliculitis, actinomyces varieties-gram-positive, anaerobic bacteria that are difficult to isolate and identify-are the most commonly isolated, although other bacteria, fungi and viruses may also appear.^1,5,10,18,19^ In contrast, there are some studies in which the most common pathogenic agent was staphylococci, followed by actinomyces.^3,13^ Because actinomyces are difficult to culture and occur in complex infections with other, easier to culture pathogens, the actinomyces growth rate reported in the literature ranges from 25 to 54%.^1,5,18,19,20^ However, it has been emphasized that actinomyces can be discovered in all cases on histopathological examination.^5,10,18^ There are case reports in which Arcanobacterium (Corynebacterium) haemolyticum (from the Actinomyces pyogenes family) grew in culture.^21^ We found actinomyces as the agent in three (75%) of the four cases we were able to analyze microbiologically, but the other three cases were using topical antibiotics when they presented to our clinic. Microbiological culture samples were not taken from these three patients due to the possible effects of the antibiotics used. However, the typical sulfur granules seen during surgery in two of the cases suggested actinomyces. In our one case of secondary canaliculitis due to a foreign body in the canaliculus, typical clinical examination findings facilitated the diagnosis.

Despite initial improvement seen with conservative treatment consisting of topical and systemic antibiotics, recurrences are common. This is thought to be due to the canalicular dacryoliths creating an obstruction that impairs tear drainage and hinders treatment penetration,^11^ which further increases the importance of early diagnosis and appropriate treatment. To repair the canaliculus, lacrimal irrigation with aqueous penicillin or povidone-iodine and sulfonamide eye drops 4 times daily, plus high-dose systemic penicillin for 3-6 months have been reported in the literature as effective against actinomyces.^20,22,23^ Briscoe et al.^5^ treated 4 Actinomyces canaliculitis patients with 20 million units/day intravenous (IV) penicillin for the first 3 weeks, followed by 3-6 months of 2 g/day oral penicillin; another 3 patients who refused IV treatment only received 2 g/day oral penicillin for 3-6 months. They found that long-term systemic penicillin was an effective treatment for Actinomyces canaliculitis. Long-term treatment has also been reported to reduce the risk of recurrence.^24^

The widely accepted treatment for canaliculitis is canaliculotomy and curettage of the canaliculus.^1,3,4,10,20^ Considering that canaliculotomy may lead to narrowing and scarring of the canalicular lumen, lacrimal pump dysfunction, and canalicular fistulas, some investigators recommend canalicular curettage alone or with the less invasive procedure of canaliculoplasty.^6,11^ However, Pavilack and Frueh^11^ reported that repeated treatment was necessary in 10 of 11 cases treated with curettage alone. In contrast, Çiftçi et al.^18^ observed recurrence in 2 of their 13 patients, and Lee et al.^6^ needed to perform repeated curettage in only 2 (6.7%) of their 30 cases, which they believed may have been the result of failure to completely remove the contents of the canaliculus. To reduce the risk of recurrence, postoperative topical and systemic antibiotic treatment is also recommended in addition to surgical treatment.^5,6,21^ Vecsei et al.,^4^ Yaman et al.^10^ and Anand et al.^3^ followed their patients for 3, 10, and a mean of 26 months, respectively, and emphasized that curettage performed with canaliculotomy was as a safe and effective treatment for canaliculitis that did not cause disruption to the canalicular or lacrimal pump systems. In order to minimize iatrogenic trauma which can lead to canalicular scarring and/or dysfunction, vertical canaliculotomy and retrograde removal of dacryoliths has been recommended as an alternative method in surgical treatment of canaliculitis.^25^ The authors performed this procedure 1 month after treatment with a 2-week course of topical antibiotic/steroid drops and oral antibiotic (doxycycline); a 2 mm vertical canaliculotomy was made, followed by the retrograde removal of the canalicular contents by medial-to-lateral pressure applied to the canaliculus with 2 cotton-tipped applicators.^25^ They reported complete clearing of the canalicular contents in their 8 patients, and observed resolution of symptoms and patency to lacrimal lavage during the follow-up period of mean 9 (range, 2-27) months.^25^

As an alternative to these surgical methods, Mohan et al.^26^ found that intracanalicular irrigation with a broad-spectrum antibiotic (50 mg/ml fortified cefazolin, 2 ml) and topical antibiotic therapy (50 mg/ml fortified cefazolin + 0.3% ciprofloxacin) were effective in the treatment of chronic suppurative canaliculitis. They reported the complete recovery of 12 patients with chronic suppurative canaliculitis using topical and intracanalicular antibiotic treatment only, without surgical intervention.^26^ Physicians sometimes encounter very rare cases of canaliculitis caused by unusual microorganisms as reported by Şen et al.^27^ where the facultative anaerobe Gemella haemolysans and anaerobe Porphyromonas asaccarolytica were determined as the causal agents. However, the patient was treated with the standard method of canaliculotomy with curettage.^27^ We used canaliculotomy and curettage in our cases and recommended the use of both oral (ampicillin/sulbactam) and topical antibiotics (100,000 U/ml penicillin) to reduce the risk of recurrence in the postoperative period. Our patients were followed up for an average of 17 (range, 3-46) months. Recurrence occurred in only one patient in the 36th month. This patient was treated with daily intracanalicular antibiotic irrigation to avoid a second surgery. Recurrence in this patient after 36 months makes us believe that a long follow-up period is necessary for canaliculitis patients. The limitations of this study are the small patient number, heterogeneous follow-up duration and its retrospective nature; however, the strength and novel contribution of this study is its emphasis on the importance of canaliculitis awareness.

## CONCLUSION

Canaliculitis should definitely be considered during the differential diagnosis of cases of recurrent, unilateral conjunctivitis in particular. Otherwise, the diagnosis may be delayed considerably, leading to incorrect treatments and even unnecessary surgical procedures such as dacryocystorhinostomy. After an accurate diagnosis, the most effective and reliable treatment is canaliculotomy with curettage. Treatment should be initiated quickly, as most patients have experienced diagnostic delays. Furthermore, culturing of the dacryoliths and discharge may facilitate better outcomes.

## Ethics

Ethics Committee Approval: It was taken, Informed Consent: It was taken.

Peer-review: External and Internal peer-reviewed.

## Figures and Tables

**Table 1:**
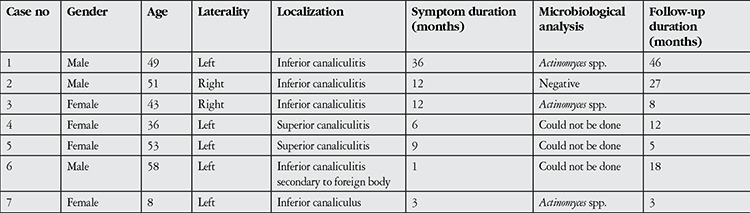
Demographic, clinical and microbiological characteristics of canaliculitis patients
